# The discovery and development of GLP-1 based drugs that have revolutionized the treatment of obesity

**DOI:** 10.1073/pnas.2415550121

**Published:** 2024-09-19

**Authors:** Jeffrey M. Friedman

**Affiliations:** ^a^HHMI at Rockefeller University, New York, NY 10065

## Abstract

The 2024 Lasker~DeBakey Clinical Medical Research Award has been given to Joel Habener and Svetlana Mojsov for their discovery of a new hormone GLP-1(7-37) and to Lotte Knudsen for her role in developing sustained acting versions of this hormone as a treatment for obesity. Each of the three had a distinct set of skills that made this advance possible; Habener is an endocrinologist and molecular biologist, Mojsov is a peptide chemist, and Knudsen is a pharmaceutical scientist. Their collective efforts have done what few thought possible—the development of highly effective medicines for reducing weight. Their research has also solved a mystery that began more than a century ago.

## The Search for Incretine

In 1906, a group in Liverpool showed that an extract from the intestine could lower blood glucose, but interest in this factor later named “incretine” waned with the discovery of insulin (reviewed in ref. 1).^*^ However, in the 1960s, interest in incretine was invigorated by the observation of three groups that orally delivered glucose increased circulating insulin concentrations and lowered blood glucose to a greater extent than did intravenously administered glucose (1). These data suggested that the gut makes a hormone(s) that regulates insulin levels, and they came to be known as incretins which were defined by Creutzfeld as factors that i) are released from gut in response to carbohydrates, ii) stimulate insulin secretion, and iii) act only in the presence of high blood glucose (2). Such a hormone could provide a novel treatment for diabetes and so the search for an incretin began. Each time a new gut peptide was identified, scientists tested its effect on insulin secretion, but only one intestinal hormone, GIP (glucose-dependent insulinotropic polypeptide), satisfied these criteria. However GIP did not show efficacy in diabetic patients so the search continued (1).

## Glucagon and the Discovery of GLP-1

“Incretine” would remain undiscovered until 1986 when Habener and Mojsov identified GLP-1(7-37) in intestine as a cleavage product of the precursor for the hormone glucagon (3, 4). Glucagon is secreted by pancreatic α cells when blood glucose is low and acts on liver to stimulate glycogenolysis, gluconeogenesis, and hepatic glucose output, thus raising glucose and preventing the sequelae of hypoglycemia. Subsequent studies using radioimmunoassays showed that glucagon-like peptides in the intestine were released in response to glucose (5), raising the possibility that a product of the glucagon gene might function as an incretin.

Joel Habener was a faculty member in the Endocrine Division at Massachusetts General Hospital investigating how hormones are produced. He decided to study the processing of the glucagon protein precursor, but instead of studying the mammalian hormone he turned his attention to fish. This was because in contrast to mammals where the islets of Langerhans are embedded in the exocrine pancreas, in fish, the islets reside in a separate organ known as Brockman bodies, thus providing an enriched source of glucagon-producing cells. Habener and colleagues made cDNAs from the Brockman bodies, and in 1982 reported that glucagon is a cleavage product of a 124 aa precursor protein that also encoded a previously unknown 34 amino acid peptide (3). This peptide sequence was preceded by two basic residues which they predicted was a cleavage site that liberated it from the precursor protein. They named the peptide glucagon-related peptide and noted that it bore significant homology to glucagon and GIP. They suggested that “biologic testing prepared either by chemical synthesis or extraction from fish islets” would be necessary.

Biologic testing using peptides prepared by chemical synthesis would soon follow. But this testing used the mammalian, not the fish form. This is because in 1983 the hamster glucagon precursor was cloned by Graeme Bell and colleagues using a degenerate oligonucleotide predicted by the glucagon sequence (6). The hamster sequence revealed clear homology between fish and hamster glucagon, but there was an important difference. The hamster glucagon-related peptide, renamed Glucagon-Like Peptide 1 or GLP-1, was 37 amino acids long with a 6 amino terminal extension not present in the fish sequence. The authors suggested that the 37-amino acid peptide homologous in fish and hamster would serve a biologic function. However, several studies failed to reveal an effect of GLP(1-37) to increase insulin secretion and its function was unclear (1).

## GLP-1 is a Prohormone

Svetlana Mojsov also studied glucagon but from an entirely different angle. In graduate school at The Rockefeller University, she joined the laboratory of Nobel laureate Bruce Merrifield, who had developed a new method for making peptides known as solid phase synthesis. In this method, a carboxy terminal amino acid was coupled to a cleavable resin after which chemically modified amino acids were sequentially added. This method was superior to the conventional method for synthesis in solutions and for her thesis Mojsov set out to synthesize glucagon with the aim of making glucagon variants that inhibited its action as potential antidiabetic agents. However, Mojsov encountered unanticipated difficulties making glucagon, owing to the low pH solutions that were used compelling her to develop an entirely new set of chemical methods for synthesizing it (7). These new chemical methods, which are still in use, provided a simple means for synthesizing a large number of glucagon variants.

But by this time, Mojsov had moved to MGH as an instructor in the Endocrine Unit and within weeks of her arrival at MGH in 1983, the Bell et al. report appeared. Mojsov was intrigued by the possibility that GLP-1 or another product of the glucagon gene might have biological effects. Soon thereafter, she was introduced to Habener and they began to collaborate with Mojsov performing the biochemical experiments and members of Habener’s laboratory conducting the molecular biology studies.

**Fig. 1.**
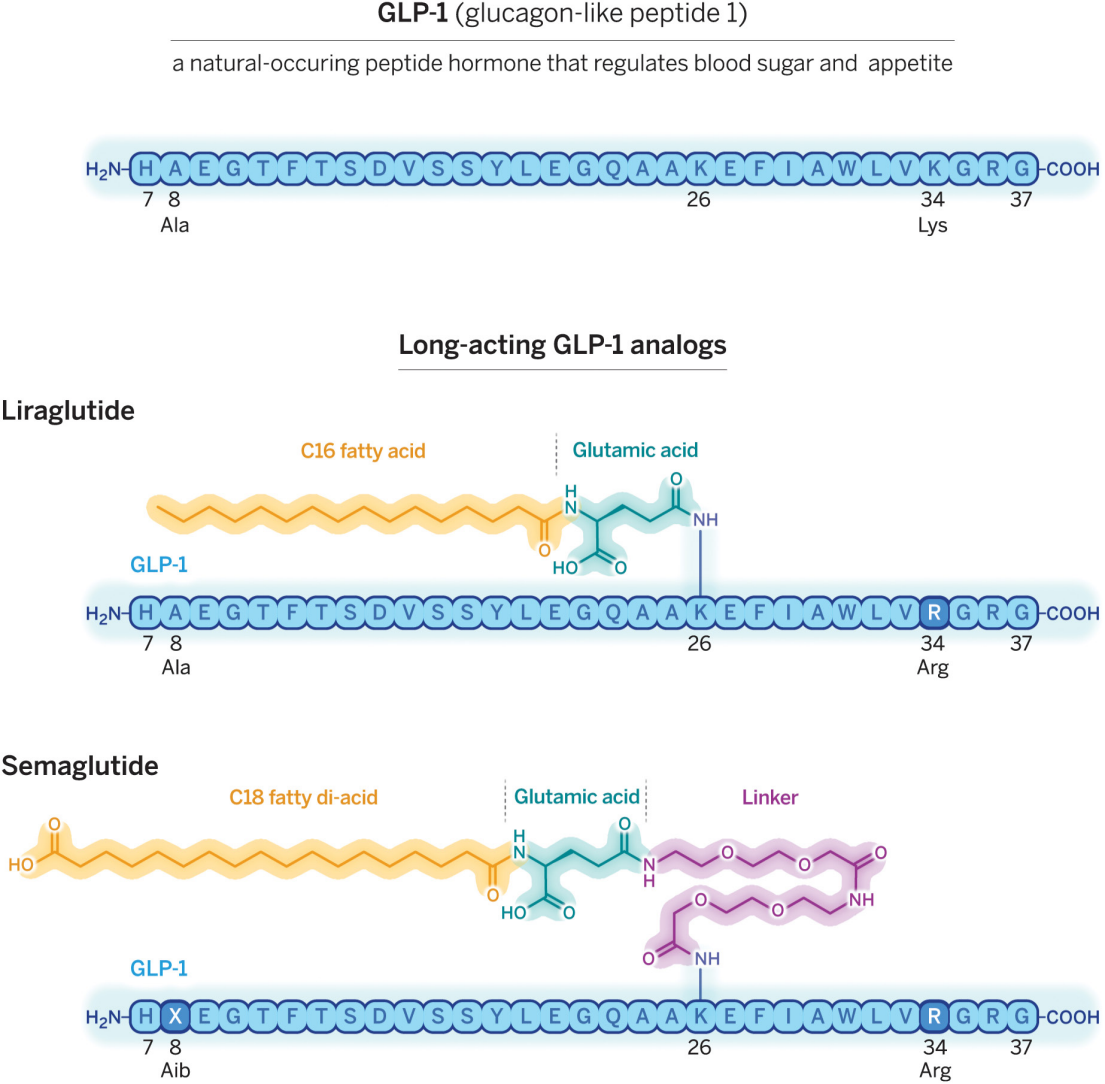
GLP-1 (*Top*) serves as the active agent in two long-acting drugs, liraglutide (*Middle*) and semaglutide (*Bottom*), that offer new hope to people who are obese or overweight. These pharmaceuticals rely on fatty acid attachments to bind the bloodborne protein albumin, which carries GLP-1 around the body and protects it from enzymatic degradation and renal clearance. To avoid immunoreactivity, both molecules hew closely to the sequence of human GLP-1. Arginine (R) replaces lysine at position 34, leaving only one lysine (K), at position 26, that can react with glutamic acid or the linker, thus ensuring that attachment occurs at the right spot. In liraglutide (*Middle*), glutamic acid connects the GLP-1 core to a fatty acid that contains 16 carbons; this drug is administered once a day. In semaglutide (*Bottom*), alpha-aminoisobutyric acid (X) in semaglutide replaces alanine (A), which protects the molecule from destruction by the enzyme DPP-4, and a long, hydrophilic linker connects an 18-carbon diacid to the GLP-1 moiety. Semaglutide is administered once a week as a subcutaneous injection. Image credit: Cassio Lynm (illustrator)/© Amino Creative.

Mojsov et al. had noted that there is a conserved histidine residue at position 7 of mammalian GLP-1(1-37) following an arginine residue and that there is greater conservation between GLP-1 and glucagon when the alignment begins there. In a notebook entry, Mojsov inserted a hand drawn arrow denoting a potential cleavage site after the arginine at position 6 (8). To test whether there was cleavage at this site, she fractionated extracts from pancreas, small intestine, and large intestine using gel filtration followed by ion exchange, assaying each by RIAs made using seven different peptides that she synthesized. She found that while there was abundant glucagon in the pancreas, only larger molecular weight forms of it were seen in intestine. As was predicted, these studies also revealed that there are large amounts of GLP-1(7-37) in the large intestine, small intestine, and pancreas of rats (Fig. 1, *Top* panel) and they also suggested that the peptide could be amidated forming GLP 7-36 amide (4).

The finding that GLP-1(7-37) is produced in intestine was a major advance and was published in September, 1986 by Mojsov et al. (4). The title of this landmark paper was “Preproglucagon Gene Expression in Pancreas and Intestine at the Level of Posttranslational Processing.” However, the key finding was, “It appears that GLP-I (1-37) may be a 'prohormone' or precursor for GLP-1 (7-37).” This rather understated conclusion encapsulates the key discovery that ultimately made the revolutionary new treatments for metabolic disease possible.

In early 1987, Mojsov, Weir, and Habener tested the insulinotropic effect of GLP-1(7-37) in the isolated rat pancreas and found it potently stimulated insulin secretion six-fold at a physiologic concentration of 5 × 10^−11^ corresponding well to its circulating levels (9). In contrast, GLP-1(1-37) had no effect even at high concentrations. Based on this, the authors suggested that GLP-1(7-37) might function as an incretin. Similar findings were also published in early 1987 by Holst et al., who confirmed that GLP-1(7-37) is present in pig small intestine using a specific RIA (10). They also showed that in the presence of glucose natural GLP-1(7-37) isolated from pig intestine or synthetic GLP-1(7-36) amide stimulated increased insulin secretion two-fold in isolated pig pancreas though only at a higher concentration of ~10^−10^ M (10). That same year, Daniel Drucker, who joined Habener’s laboratory as a postdoctoral fellow in 1984—together with Mojsov and Habener—also reported that GLP-1(7-37) stimulated insulin secretion from an islet cell line in a glucose-dependent manner though only at an even higher concentration of 5 × 10^−7^ M (11).

In 1987 in a study of seven human volunteers, Bloom et al. showed that an infusion of GLP-1(7-36) increased plasma insulin and reduced plasma glucose in patients that received a glucose load but not in patients who were fasted. GLP-1(7-37) and GLP-1(7-36) amide both circulate in similar amounts and have equivalent bioactivity. With these aggregate findings, GLP-1(7-37) now satisfied all of the criteria for an incretin (12). However, it was not clear whether the hormone would provide a clinical benefit in diabetic patients.

In 1992 and 1993, several reports confirmed a clinical response in diabetic patients, including a study by David Nathans, Mojsov, and Habener, who showed that a 30-min intravenous infusion of GLP-1(7-37) significantly increased insulin levels and lowered glucose levels in diabetic patients, the latter of whom showed a 3-fold increase in insulin (13). Similar findings were reported by groups led by Efendic et al. (14) and Nauck et al. (15). These pivotal results established the potential of this new hormone as a therapy for diabetes but there was a problem. The half-life of GLP-1(7-37) in plasma is 1 to 2 min, necessitating the use of an infusion to deliver it in sufficient amounts. The formidable challenge was now to engineer a stable version of GLP-1(7-37). At this point, Lotte Knudsen, a young scientist at Novo Nordisk, entered the scene.

## An Effective New Treatment for Metabolic Disease

Since its founding, Novo Nordisk has focused on making different forms of insulin, ultimately becoming the world's largest producer. However, in the early 1990s, Novo formed an innovation team to explore new approaches. Knudsen, who was working in the chemical division, was invited to join this new group and by 1995 she was leading it. One of their priorities was to develop GLP-1(7-37) into a drug. From the outset, Knudsen believed that this would not only become a treatment for diabetes but one for obesity. This was because several studies from Ole Madsen (University of Copenhagen) had shown that transplantation of some glucagon producing tumors caused profound anorexia in animals (16). Consistent with this, Bloom et al. had also shown that intracerebroventricular injections of GLP-1(7-36) amide into rats profoundly reduced food intake (17).

Initially, the team at Novo considered several different approaches, but opted to make a more stable version of GLP-1(7-37). For daily administration, they would need to modify the molecule so that it would have a half-life of ~10 h instead of the 1 to 2 min half-life of the native hormone which is both degraded in plasma and rapidly filtered by the kidney. The modified drug would need to remain active 24 h/d, be as or more potent than native GLP-1, and remain physically stable for long periods of time. To achieve this, Knudsen and her team decided to use a novel approach first developed by chemists at Novo in which a fatty acid is attached to a lysine (or other) residue via a linker (18). The modification causes it to bind to albumin, protecting it from metabolic degradation and decreasing its renal clearance, thus prolonging its half-life.

Knudsen’s group used mutagenesis to find critical residues and then tested different fatty acids, linkers, and coupling sites. In 2000, they reported the properties of liraglutide, a GLP-1(7-37) derivative with a C16 fatty acid coupled to a lysine at position 26 (19). Lysine 34 was also mutated to arginine to prevent fatty acid coupling there (Fig. 1, *Middle* panel). This increased the half-life to 13 h and had the desired product profile. Clinical studies began shortly thereafter, but in the late 1990s, their program encountered several obstacles. Some patients reported nausea, leading Novo to reduce the dose, but efficacy was disappointing. And by now, there was competition from two companies pursuing alternative approaches that Novo had previously deprioritized.

In the mid 1980s, John Eng was studying bioactive peptides from the venom of the Gila monster by screening for molecules with an amino-terminal histidine which he noted was shared by GLP-1 and other gut peptides. He identified a novel peptide named Exendin-4 which had extensive homology to GLP-1(7-37) and competed with its binding to the GLP-1 receptor and stimulated insulin secretion in rodents (1, 20). Importantly Exendin-4 has a mutation that blocks its cleavage extending its half-life to 2.4 h. Eng then began a collaboration with Amylin Pharmaceutical and in a series of clinical trials, exendin-4 injected twice daily showed highly significant efficacy in Type 2 diabetic patients with lowering of HbA1c by 0.86% and modest weight loss of 1.6 kg (1, 20). Exenatide was approved for treatment of diabetes in 2004.

Other companies began developing drugs that inhibit DPP-4, the enzyme that degrades GLP-1(7-37). Sitagliptin, the first approved DPP-4 inhibitor, was developed by a group led by Nancy Thornberry and Ann Weber at Merck and lowered HbA1c by ~0.8% as a monotherapy with a highly significant reduction of ~1.9% when icombined with metformin (1). Sitagliptin was approved in 2006, but in contrast to the GLP-1 agonists, DPP4 inhibitors have little or no effect on body weight.

Despite the aforementioned hurdles and the brewing competition, Knudsen’s Novo team pressed on. A key insight was the realization that the nausea seen after injections of liraglutide (Victoza) could be mitigated if one started at a lower dose and then gradually increased it. Using a protocol of escalating doses, a once daily injection significantly decreased HbA1c by 1.1 to 1.6% in diabetic patients and also caused significant weight loss of 5% or greater in 54% of patients, leading to its approval in 2010 (21). Further, despite internal resistance at Novo, Knudsen initiated trials administering a higher dose of liraglutide (Saxenda) for the treatment of obesity. This resulted in an average of ~5 kg weight loss with the majority of patients losing more than 5% of their weight (1). However, this was just the beginning.

## A Highly Effective Treatment for Obesity

To further compliance, Knudsen and a team of Novo chemists led by Jesper Lau and Thomas Kruse set out to make a GLP-1 receptor agonist that could be administered once weekly (22). They tested thousands of different combinations of linkers, fatty acids along with changes the amino acid sequence leading to the development of semaglutide, an ultrastable receptor agonist in which a carboxylated fatty acid is coupled to lysine 26 via a hydrophilic linker (Fig. 1, *Bottom* panel). The molecule also has a mutation that prevents DPP-4 cleavage. These modifications—not present in liraglutide—result in an extension of its half-life to 7 d and weekly subcutaneous administration led to an HBA1c reduction of 1.9%.

There was, however, a surprise. Diabetic patients treated with semaglutide (Ozempic) showed dose-dependent weight loss with an average ~6 kg at a 2 mg dose with 40% of patients losing more than 10% of their weight (1). This was followed by clinical trials for the treatment of obesity using higher, escalating doses of the drug (Wegovy). The results were stunning. Treated patients lost an average of 12.4% of their initial body weight compared to individuals who received placebo (1). The health implications of these results are profound (1). In diabetic patients, semaglutide significantly reduced the incidence of cardiovascular events by 26% vs. placebo (23). Weekly semaglutide also reduced the incidence cardiovascular events in overweight patients with pre-existing cardiovascular disease but without diabetes and also in obese patients without diabetes (24). The GLP-1 receptor is broadly distributed in the periphery and brain, raising the possibility that there could be additional unanticipated health benefits. For example, recent studies have reported clinical benefits in patients with heart and kidney failure. The potential benefit of semaglutide in other clinical settings is being actively explored.

While semaglutide thus far appears to be quite safe, the nausea that it causes is clinically significant, leading many patients to discontinue treatment (1). GLP-1RAs also delay gastric emptying and there are reports that many surgeons recommend discontinuing it temporarily prior to surgery. Semagutide needs to be taken continuously to be effective and treatment results in loss of both fat and muscle mass, which appears to be equivalent to that seen after weight loss by dieting (1). However, muscle mass does not appear to recover when the drug is stopped and is of potential concern especially for older patients though its impact on overall health is unclear (1). These agents have now been in patients for decades without major safety signals but as with any agent, further vigilance is required.

## The Future

The proven health benefits of GLP-1 receptor agonists (GLPRAs), like liraglutide (Saxenda®) and semaglutide (Wegovy®), have transformed the treatment of metabolic disease and its discovery has stimulated development of other GLP-1(7-37)-based therapeutic approaches, including an oral GLPRA, which has shown efficacy (1). GLPRAs have also shown enhanced efficacy when combined with agonist activity at other peptide hormone receptors. An approach pioneered by Matthias Tschoep and Richard DiMarchi identified even more potent agents that activate the GLP-1 receptor together with other gut hormone receptors (1). These include a dual agonist combining glucagon and GLP-1 activity and another with GIP and GLP-1 activity. In 2022, a modified version of the GLP-1/GIP dual agonist, tirzepatide (Zepbound®), developed by Eli Lilly induced an average weight loss of 21% in humans with obesity and 15% weight loss in obese patients with diabetes (1). The enhanced efficacy of dual glucagon-R/GLP-1R and GIP-R/GLP-1R coagonists also led Tschoep and DiMarchi to make a unimolecular triple coagonist with activity for the receptors for GIP, glucagon and GLP-1. Phase 2 data of retatrutide, a similar triple agonist developed by Eli Lilly, has shown a remarkable 24% weight loss after 48 wk of treatment, without reaching a plateau (1).

## Summary

The cloning of the glucagon gene, identification of GLP-1(7-37) as an intestinal hormone satisfying the criteria of an incretin and its development into a drug is a milestone in medicine. These achievements have solved a more than hundred-year-old mystery about the role of the gut in glucose metabolism, identified a new endocrine mechanism regulating insulin secretion and provided the first effective treatment for obesity with profound implications for maintaining human health. This year's Lasker~DeBakey Clinical Medical Research Award recognizes Joel Habener, Svetlana Mojsov, and Lotte Knudsen for their essential roles in making this possible.

## Data Availability

There are no data underlying this work.

## Footnotes

^*^Due to space limitations several publications were not cited, and in such instances readers are directed to a review article, citation (1), in which they were referenced.
